# Incidence of childhood CNS tumours in Britain and variation in rates by definition of malignant behaviour: population-based study

**DOI:** 10.1186/s12885-019-5344-7

**Published:** 2019-02-11

**Authors:** Charles A. Stiller, Anita M. Bayne, Aruna Chakrabarty, Tom Kenny, Paul Chumas

**Affiliations:** 1 National Cancer Registration and Analysis Service, Public Health England, 4150 Chancellor Court, Oxford Business Park South, Oxford, OX4 2GX UK; 2 Histopathology, Leeds Teaching Hospitals NHS Trust, Leeds, UK; 3 Faculty of Health & Social Sciences, University of Bournemouth, Bournemouth, UK; 4 Department of Neurosurgery, Leeds Teaching Hospitals NHS Trust, Leeds General Infirmary, Leeds, UK

**Keywords:** CNS tumours, Childhood, Incidence, Behaviour codes, Trends

## Abstract

**Background:**

Intracranial and intraspinal tumours are the most numerous solid tumours in children. Some recently defined subtypes are relatively frequent in childhood. Many cancer registries routinely ascertain CNS tumours of all behaviours, while others only cover malignant neoplasms. Some behaviour codes have changed between revisions of the International Classification of Diseases for Oncology, including pilocytic astrocytoma, downgraded to uncertain behaviour in ICD-O-3.

**Methods:**

We used data from the population-based National Registry of Childhood Tumours, which routinely included non-malignant CNS tumours, to document the occurrence of CNS tumours among children aged < 15 years in Great Britain during 2001–2010 and to document the descriptive epidemiology of childhood CNS tumours over the 40-year period 1971–2010, during which several new entities were accommodated in successive editions of the WHO Classification and revisions of ICD-O. Eligible cases were all those with a diagnosis included in Groups III (CNS tumours) and Xa (CNS germ-cell tumours) of the International Classification of Childhood Cancer, Third Edition. The population at risk was derived from annual mid-year estimates by sex and single year of age compiled by the Office for National Statistics and its predecessors. Incidence rates were calculated for age groups 0, 1–4, 5–9 and 10–14 years, and age-standardised rates were calculated using the weights of the world standard population.

**Results:**

Age-standardised incidence in 2001–10 was 40.1 per million. Astrocytomas accounted for 41%, embryonal tumours for 17%, other gliomas for 10%, ependymomas for 7%, rarer subtypes for 20% and unspecified tumours for 5%. Incidence of tumours classified as malignant and non-malignant by ICD-O-3 increased by 30 and 137% respectively between 1971-75 and 2006–10.

**Conclusions:**

Total incidence was similar to that in other large western countries. Deficits of some, predominantly low-grade, tumours or differences in their age distribution compared with the United States and Nordic countries are compatible with delayed diagnosis. Complete registration regardless of tumour behaviour is essential for assessing burden of disease and changes over time. This is particularly important for pilocytic astrocytoma, because of its recent downgrading to non-malignant and time trends in the proportion of astrocytomas with specified subtype.

## Background

Intracranial and intraspinal tumours are the most frequent solid tumours in children. Age-standardised incidence at age 0–14 years in industrialised countries during the 1980s–90s was typically in the range 30–40 per million [1, 2]. Successive standard classifications of childhood cancer, while generally restricted to neoplasms of malignant behaviour, have also admitted non-malignant tumours of the brain, spinal cord, meninges and intracranial endocrine glands because benign tumours of these sites are potentially lethal and it can be peculiarly difficult to determine the behaviour of some tumours, especially in the absence of histology [3–6]. Many population-based general cancer registries, and all specialist children’s cancer registries, have for a long time routinely ascertained these non-malignant tumours for the same reasons. Other registries traditionally confined their coverage to malignant neoplasms, however, including most of those in the United States until they were mandated to include non-malignant CNS tumours from 2004 onwards. In the interests of comparability, successive volumes of *Cancer Incidence in Five Continents* have always been restricted to malignant neoplasms [7, 8]. By contrast, the two volumes of *International Incidence of Childhood Cancer* [1, 9] and the Automated Childhood Cancer Information System [2] included intracranial and intraspinal tumours regardless of behaviour, but provided information on the proportion specified as malignant.

In the past three decades, new types of intracranial and intraspinal neoplasms have been defined, including several that are relatively frequent in childhood [10–13] (Tables 1 and 2). Some are non-malignant tumours that would previously have been included within a less specific entity that was regarded as malignant. Some entities changed behaviour code between revisions of the International Classification of Diseases for Oncology (ICD-O) [14–16] (Table 1). Mostly these affected only small numbers of cases, but pilocytic or juvenile astrocytoma, the most frequent of all childhood central nervous system (CNS) tumours, was downgraded from malignant to uncertain behaviour in ICD-O-3, published in 2000 [16].

**Table 1:** Changes and additions to morphology codes for intracranial and intraspinal tumours between ICD-O-1 (1976) [14], ICD-O-2 (1990) [15] and ICD-O-3 (2000) [16]. Changes to behaviour code are in ***bold italics***. ICCC-3: category in International Classification of Childhood Cancer, Third Edition [6]

Entity	ICD-O-1	ICD-O-2	ICD-O-3	ICCC-3
Papillary ependymoma	9393/1	9393/1	***9393/3***	IIIa.1
Pilocytic astrocytoma	9421/3	9421/3	***9421/1***	IIIb
Spongioblastoma, NOS	9422/3	9422/3	***9421/1***	IIIb
Pleomorphic xanthoastrocytoma	–	9424/3	9424/3	IIIb
Gliofibroma	–	–	9442/1	IIIb
Large cell medulloblastoma	–	–	9474/3	IIIc.1
Primitive neuroectodermal tumour, NOS (now Embryonal CNS tumour, NOS)	–	9473/3	9473/3	IIIc.2
Atypical teratoid/rhabdoid tumour	–	8963/3	9508/3	IIIc.4
Chordoid glioma	–	–	9444/1	IIId.3
Prolactinoma	–	8271/0	8271/0	IIIe.1
Pituitary adenoma, NOS	8140/0	8140/0	8272/0	IIIe.1
Pituitary carcinoma, NOS	8010/3	8010/3	8272/3	IIIe.1
Adamantinomatous craniopharyngioma	–	–	9351/1	IIIe.2
Papillary craniopharyngioma	–	–	9352/1	IIIe.2
Granular cell tumour of the sellar region	9580/0	9580/0	9582/0	IIIe.2
Desmoplastic infantile astrocytoma	–	–	9412/1	IIIe.4
Dysembryoplastic neuroepithelial tumour	–	–	9413/0	IIIe.4
Gangliocytoma	–	9490/0	9492/0	IIIe.4
Dysplastic gangliocytoma of cerebellum	–	–	9493/0	IIIe.4
Anaplastic ganglioglioma	–	–	9505/3	IIIe.4
Clear cell meningioma			9538/1	IIIe.5
Papillary meningioma	9538/1	9538/1	***9538/3***	IIIe.5

**Table 2:** Additional ICD-O morphology codes proposed in the 2007 WHO Classification of Tumours of the Central Nervous System and included in ICD-O-3.1 [19], with provisional allocation to categories in ICCC-3

Code	Entity	ICCC-3
9390/1	Atypical choroid plexus papilloma	IIIa.2
9395/3	Papillary tumour of the pineal region	IIId.3
9425/3	Pilomyxoid astrocytoma	IIIb
9431/1	Angiocentric glioma	IIId.3
9432/1	Pituicytoma	IIId.3
9509/1	Papillary glioneural tumour/rosette-forming glioneuronal tumour of the fourth ventricle	IIIe.4

There are few detailed accounts of the occurrence of childhood CNS tumours based on large series from population-based cancer registries using data coded to ICD-O-3 [17, 18].

This study has two principal aims. The first is to give a detailed and up to date account of the occurrence of childhood intracranial and intraspinal tumours based on data from a large, population-based, specialist children’s tumour registry, including information on subtypes that were not assigned specific morphology codes until ICD-O-3. The second is to use data from the same registry to document the descriptive epidemiology of childhood CNS tumours over a longer period during which new entities were accommodated in successive editions of the WHO Classification [10–12] and revisions of ICD-O [15, 16, 19].

## Methods

The population-based National Registry of Childhood Tumours (NRCT) covered the population under 15 years of age throughout Great Britain (England, Scotland and Wales) during 1962–2010. Sources of notification varied slightly over the years but always included the network of regional and national cancer registries covering the population of all ages in the whole of Great Britain, together with death certificates for children with a neoplasm as underlying cause of death. Cases were also ascertained from specialist children’s tumour registries in certain regions of England. Since 1977 increasing numbers were notified directly from paediatric oncology principal treatment centres [20]. The NRCT always included all malignant neoplasms and all non-malignant intracranial and intraspinal tumours. Diagnoses were coded according to ICD-O [14–16], with earlier data recoded retrospectively on adoption of each new revision. Further details of the NRCT are published elsewhere [21]. Diagnoses for this study were coded to ICD-O-3 [16] and grouped by the International Classification of Childhood Cancer, Third Edition (ICCC-3) [6]. Additional codes included in the first revision of ICD-O-3 [19] were provisionally allocated to ICCC-3 categories (Table 2). Definitions for four existing codes were expanded in line with the 2007 WHO Classification: 8290/0 includes spindle-cell oncocytoma, 9471/3 includes medulloblastoma with extensive nodularity, 9474/3 includes anaplastic medulloblastoma, and 9506/1 includes extraventricular neurocytoma and cerebellar liponeurocytoma [12, 19]. Additional entities in the 2016 WHO Classification, many of them genetically defined, could not be identified in the registry data, but we have adopted the preferred terminology ‘embryonal CNS tumour NOS’ to replace ‘primitive neuroectodermal tumour (PNET)’ [13].

For this study we included all children who were under the age of 15 years and resident in Great Britain when diagnosed with a tumour included in ICCC-3 Group III ‘Central nervous system and miscellaneous intracranial and intraspinal neoplasms’ or Subgroup Xa ‘Intracranial and intraspinal germ-cell tumours’ during 1971–2010. The main analyses are restricted to cases of a first or only tumour in these ICCC-3 categories, but brief details are also given of the small number of tumours diagnosed in children with a previous cancer diagnosis where it was clear from diagnostic and other reports that these were distinct primary tumours. The detailed description of incidence is based on children diagnosed during 2001–10. Data from the full 40-year study period were used to describe changes in recorded incidence and relative frequency over time and to show the effects of restricting coverage to cases defined as malignant by successive revisions of ICD-O.

The population at risk was derived from annual mid-year estimates by sex and single year of age compiled by the Office for National Statistics and its predecessors. The average population at risk was 10.5 million children during 2001–10 and 11.1 million children during 1971–2010.

Registration rates were calculated per million child-years at risk for age groups 0, 1–4, 5–9 and 10–14 years [1]. Age-standardised rates (ASR) were calculated by the direct method, using the world standard population which assigns weights 2.4, 9.6, 10 and 9 respectively to these age groups [1]. Cumulative risk to 15th birthday was defined as the reciprocal of the cumulative rate which was calculated as r_0_ + 4r_1_ + 5r_2_ + 5r_3_, where r_0_, r_1_, r_2_ and r_3_ are the age-specific rates for ages 0, 1–4, 5–9 and 10–14 respectively [1].

## Results

### Incidence 2001–2010

Table 3 shows numbers of registrations for children diagnosed during 2001–10, percentage recorded as microscopically verified (MV), annual rates per million children by age group, and ASRs by sex and for both sexes combined. Data are shown for ICCC-3 subgroups and divisions and the principal subtypes within them. A total of 4166 tumours were registered, 2275 in boys and 1891 in girls. Numbers by age group were: age 0, 287; age 1–4, 1213; age 5–9, 1320; age 10–14, 1346. Overall, 79% of cases were recorded as MV. The % MV exceeded 90% for many diagnostic subgroups and was over 80% for the great majority but was only 8% for unspecified optic nerve glioma/astrocytoma, 18% for unspecified glioma of other sites and 20% for unspecified tumours. The total ASR was 40.1 per million.

**Table 3:** Intracranial and intraspinal tumours among children aged 0–14 years resident in Great Britain, 2001–2010. Numbers of registrations (N), % microscopically verified (MV), rates per million children by age group, age standardised rates (world standard population) (ASR) by sex

									**ASR**		
**ICCC-3**			**N**	**%MV**	**0**	**1–4**	**5–9**	**10–14**	**M**	**F**	**Total**
IIIa.1	Ependymoma		291	96.9	2.6	4.4	2.4	1.9	3.1	2.7	2.9
		Unspecified	149	96.0	1.4	2.2	1.4	0.9	1.6	1.4	1.5
		Anaplastic/ependymoblastoma	109	100	1.1	2.1	0.8	0.4	1.2	1.0	1.1
		Papillary	3	66.7	–	< 0.05	–	0.1	< 0.05	< 0.05	< 0.05
		Myxopapillary	28	100	–	0.1	0.2	0.5	0.3	0.2	0.2
		Subependymoma	2	0	–	–	< 0.05	< 0.05	–	< 0.05	< 0.05
IIIa.2	Choroid plexus tumours		111	99.1	6.4	1.5	0.4	0.3	1.3	1.1	1.2
		Papilloma	62	100	3.4	0.8	0.2	0.2	0.8	0.5	0.7
		Atypical papilloma	13	100	1.0	0.1	0.1	–	0.1	0.2	0.1
		Carcinoma	36	97.2	2.0	0.6	0.1	0.1	0.4	0.4	0.4
IIIb	Astrocytoma		1707	81.7	11.1	19.4	16.5	14.7	16.4	16.5	16.5
		Unspecified (optic nerve)	249	7.6	1.7	4.5	2.3	1.0	2.3	2.8	2.5
		Unspecified (other sites)	222	82.9	1.3	2.5	2.3	1.9	2.3	2.0	2.1
		Pilocytic	860	97.0	2.6	9.7	8.5	7.9	8.0	8.4	8.2
		Subependymal giant cell	42	78.6	0.4	0.2	0.4	0.5	0.5	0.3	0.4
		Gliofibroma	1	100	–	–	–	< 0.05	–	< 0.05	< 0.05
		Protoplasmic	1	100	–	< 0.05	–	–	–	< 0.05	< 0.05
		Gemistocytic	3	100	–	–	–	0.1	< 0.05	–	< 0.05
		Fibrillary	29	96.6	–	0.4	0.1	0.3	0.3	0.2	0.3
		Pleomorphic xanthoastrocytoma	22	100	–	0.1	0.1	0.4	0.2	0.2	0.2
		Pilomyxoid	38	97.4	1.6	0.5	0.1	0.2	0.4	0.4	0.4
		Anaplastic	93	97.8	1.0	0.6	1.0	0.9	0.9	0.8	0.9
		Glioblastoma	147	97.3	2.6	0.8	1.7	1.3	1.5	1.3	1.4
IIIc.1	Medulloblastoma		522	98.5	3.3	5.9	6.1	3.6	6.5	3.5	5.1
		Unspecified	394	98.0	2.0	3.9	5.0	2.8	5.0	2.5	3.8
		Desmoplastic/nodular/extensive nodularity	84	100	1.1	1.5	0.5	0.5	1.0	0.7	0.9
		Medullomyoblastoma	6	100	–	0.1	0.1	–	0.1	–	0.1
		Large cell/anaplastic	38	100	0.1	0.4	0.5	0.2	0.4	0.3	0.4
IIIc.2	Embryonal CNS tumour NOS		111	95.5	2.7	1.6	0.7	0.7	1.0	1.2	1.1
IIIc.3	Medulloepithelioma/neuroepithelioma		3	100	–	< 0.05	0.1	–	< 0.05	< 0.05	< 0.05
IIIc.4	Atypical teratoid/rhabdoid tumour		81	96.3	3.3	1.7	0.2	0.2	0.9	0.9	0.9
IIId.1	Oligodendroglioma		34	100	–	0.5	0.3	0.3	0.3	0.3	0.3
IIId.2	Mixed and unspecified gliomas		340	22.9	1.0	3.2	4.2	2.8	3.4	3.1	3.2
		Unspecified	318	17.6	1.0	3.0	4.1	2.4	3.2	2.9	3.0
		Mixed	19	100	–	0.1	0.1	0.4	0.2	0.2	0.2
		Angiocentric glioma	3	100	–	< 0.05	< 0.05	< 0.05	< 0.05	< 0.05	< 0.05
IIId.3	Other neuroepithelial tumours		23	82.6	0.1	0.1	0.2	0.3	0.2	0.2	0.2
		Gliomatosis cerebri	13	76.9	0.1	0.1	0.1	0.2	0.2	0.1	0.1
		Papillary tumour of the pineal region	5	100	–	–	0.1	0.1	< 0.05	0.1	< 0.05
		Astroblastoma	5	80.0	–	0.1	< 0.05	0.1	< 0.05	0.1	< 0.05
IIIe.1	Pituitary adenoma		67	62.7	–	0.1	0.2	1.5	0.5	0.6	0.6
IIIe.2	Craniopharyngioma		191	82.7	0.1	1.2	2.2	2.2	1.8	1.7	1.7
IIIe.3	Pineal parenchymal tumours		46	97.8	0.4	0.4	0.4	0.4	0.4	0.4	0.4
		Pineocytoma	4	100	–	< 0.05	0.1	< 0.05	< 0.05	< 0.05	< 0.05
		Pineoblastoma incl. PTID	42	97.6	0.4	0.4	0.4	0.4	0.4	0.4	0.4
IIIe.4	Mixed glial-neuronal tumours		223	91.5	3.1	1.6	1.9	2.6	2.3	1.8	2.1
		Desmoplastic infantile astrocytoma	21	100	2.1	0.1	0.1	< 0.05	0.3	0.1	0.2
		Dysembryoplastic neuroepithelial tumour	91	86.8	0.1	0.7	0.9	1.1	1.0	0.7	0.8
		Ganglioglioma	96	93.8	0.6	0.7	0.8	1.3	0.9	0.9	0.9
		Central neurocytoma	6	83.3	0.1	0.1	< 0.05	0.1	0.1	–	0.1
		Papillary glioneuronal tumour	3	100	–	–	0.1	< 0.05	< 0.05	< 0.05	< 0.05
		Gangliocytoma	6	100	0.1	0.1	0.1	< 0.05	< 0.05	0.1	0.1
IIIe.5	Meningioma		50	88.0	0.1	0.3	0.3	0.8	0.4	0.5	0.4
IIIf	Unspecified tumours		196	19.9	4.5	1.6	1.5	1.9	2.0	1.8	1.9
Xa	Germ cell tumours		170	77.6	2.0	0.7	1.0	2.9	1.9	1.1	1.5
		Germinoma	119	76.5	0.1	< 0.05	0.8	2.4	1.3	0.7	1.0
		Other	51	80.4	1.8	0.7	0.1	0.4	0.6	0.4	0.5
		Total	4166	78.8	40.7	44.4	38.6	37.0	42.6	37.5	40.1

Source: National Registry of Childhood Tumours

Ependymomas accounted for 7% of registrations. This subgroup included ependymoblastoma, although it is usually regarded as a type of embryonal tumour, because it shares a morphology code with the more frequently occurring anaplastic ependymoma. Anaplastic ependymomas and ependymoblastoma accounted for 37% of ependymomas, myxopapillary ependymoma for 10% and other specified subtypes for 2%; 51% were of unspecified subtype. Ependymoma was generally most frequent at age 1–4 years, but 68% of myxopapillary ependymomas were diagnosed at age 10–14. Choroid plexus tumours accounted for 3% of registrations. Of these, 56% were benign or unspecified papillomas, 12% were atypical choroid plexus papillomas and 32% were carcinomas. All types of choroid plexus tumour were most often seen in the first year of life.

Astrocytomas were the largest diagnostic subgroup, 41% of all registrations. Fifty percent of all astrocytomas were pilocytic. Subependymal giant cell astrocytoma (SEGA), fibrillary astrocytoma and pilomyxoid astrocytoma each accounted for around 2%, pleomorphic xanthoastrocytoma for 1% and other rare low-grade (WHO Grade I and II) subtypes for 0.3%; 5% of astrocytomas were anaplastic, 9% were glioblastoma and 28% were of unspecified subtype. Among the 471 unspecified astrocytomas, 249 (53%) were optic nerve gliomas. If all optic nerve gliomas are assumed to have been low-grade, 73% of all astrocytomas were low-grade, 14% were high-grade (WHO Grade III-IV) and 13% were of unspecified grade. Low-grade astrocytomas predominated in all age groups. The highest incidence of pilocytic astrocytoma and unspecified optic nerve astrocytoma was at age 1–4 years. Pleomorphic xanthoastrocytoma was exceedingly rare before age 10 years, whereas by far the highest incidence of pilomyxoid astrocytoma was in the first year of life. For both subtypes of high-grade astrocytoma, anaplastic astrocytoma and glioblastoma, there was an early peak of incidence in the first year of life, a nadir at age 1–4 and somewhat higher incidence at age 5 years and over.

Other gliomas accounted for 10% of registrations. The great majority, 80%, were of unspecified subtype. Nearly all the remainder were oligodendrogliomas (9%), mixed gliomas (5%) or gliomatosis cerebri (3%). Incidence was highest at age 5–9 years.

Embryonal tumours accounted for 17% of registrations. Within this subgroup, 73% were medulloblastoma, 15% were embryonal CNS tumour NOS and 11% were atypical teratoid/rhabdoid tumours (ATRT). Incidence of medulloblastoma was highest at age 1–9 years, whereas both embryonal CNS tumour NOS and ATRT were most frequent in the first year of life. Three quarters of medulloblastoma were of classical or unspecified subtype, 16% were desmoplastic/nodular or with extensive nodularity, and 7% were large-cell/anaplastic. Incidence of the desmoplastic/nodular subtype was highest at 1–4 years of age whereas the age distribution of the large-cell/anaplastic subtypes was similar to that of classical/unspecified medulloblastoma, with highest incidence at age 5–9 years.

The subgroup of other specified tumours, excluding germ-cell tumours, accounted for 14% of registrations. Mixed neuronal-glial tumours represented 39% of the subgroup; within this category, 43% were gangliogliomas, 41% were dysembryoplastic neuroepithelial tumours (DNET), and 9% were desmoplastic infantile astrocytoma (DIA). Tumours of the sellar region (craniopharyngiomas) represented 33% of the subgroup, pituitary adenomas 12%, pineal parenchymal tumours 8% and meningiomas 9%. Of the craniopharyngiomas, 41% were adamantinomatous, one (< 1%) was papillary, and 58% were of unspecified subtype. Among pituitary adenomas, 33% were prolactinomas, 9% were basophil adenomas and 58% were unspecified.

Germ-cell tumours accounted for 4% of registrations. They were mostly germinomas (70%) or teratomas (25%). Germinomas occurred predominantly in the 10–14 age group whereas teratomas were most frequent in infancy.

Finally, 5% of registrations were for tumours of unspecified type, mostly without microscopic verification.

The great majority (4007, 96%) of tumours in this series were intracranial, but there were also 159 intraspinal tumours, accounting for 3.8% and with an ASR of 1.6 per million. They comprised intramedullary and extramedullary intradural tumours, together with extradural ependymomas since the latter are included in ICCC-3 Subgroup IIIa and occur at midline sites below the head. The most frequent types of intraspinal tumour were astrocytoma (47%) and ependymoma (26%).

### Incidence 1971–2010

There were 14,281 registrations during the entire 40-year study period. Table 4 presents numbers of cases and ASRs in successive 5-year periods, classified by ICCC-3 and behaviour as defined by ICD-O-3. Within subgroup IIIb, astrocytomas, pilocytic astrocytoma is shown separately and registrations are also subdivided by site (optic nerve and other) and ICD-O-3 behaviour.

**Table 4:** Intracranial and intraspinal tumours among children aged 0–14 years resident in Great Britain, 1971–2010. Numbers of registrations (N) and age-standardised rates (world standard population) (ASR) in successive quinquennia of diagnosis, by ICCC-3 category and ICD-O-3 behaviour code

ICCC-3		1971–1975		1976–1980		1981–1985		1986–1990		1991–1995		1996–2000		2001–2005		2006–2010	
		N	ASR	N	ASR	N	ASR	N	ASR	N	ASR	N	ASR	N	ASR	N	ASR
IIIa.1	Ependymoma	181	2.9	164	3.0	131	2.6	121	2.4	127	2.4	134	2.6	139	2.8	152	3.0
IIIa.2	Choroid plexus tumours	13	0.2	24	0.5	31	0.7	28	0.6	43	0.9	48	1.0	54	1.2	57	1.2
IIIb	Astrocytoma	563	8.7	611	10.3	518	9.7	606	11.8	780	14.4	786	14.5	842	16.1	865	16.8
	Pilocytic, optic nerve	12	0.2	16	0.3	10	0.2	15	0.3	25	0.5	22	0.4	31	0.7	29	0.6
	Pilocytic, other sites	173	2.7	222	3.7	162	3.0	181	3.5	256	4.7	360	6.6	382	7.2	418	8.0
	Other non-malignant behaviour	2	< 0.05	5	0.1	3	< 0.05	5	0.1	13	0.2	23	0.4	21	0.4	21	0.4
	Malignant behaviour, optic nerve	43	0.7	49	0.9	46	0.9	52	1.1	71	1.4	102	2.0	113	2.3	139	2.8
	Malignant behaviour, other sites	333	5.1	319	5.3	297	5.5	353	6.8	415	7.6	279	5.1	295	5.5	258	5.0
IIIc.1	Medulloblastoma	316	5.0	306	5.3	277	5.4	237	4.6	256	4.8	254	4.7	272	5.3	252	4.9
IIIc.2	Embryonal CNS tumour NOS	2	< 0.05	9	0.2	40	0.8	70	1.4	86	1.7	75	1.5	50	1.0	59	1.2
IIIc.3	Medulloepithelioma/neuroepithelioma	1	< 0.05	2	< 0.05	0	–	2	< 0.05	5	0.1	2	< 0.05	2	< 0.05	1	< 0.05
IIIc.4	Atypical teratoid/rhabdoid tumour	0	–	0	–	0	–	2	< 0.05	2	< 0.05	22	0.5	32	0.7	49	1.0
IIId.1	Oligodendroglioma	34	0.5	21	0.3	33	0.6	24	0.5	19	0.3	13	0.2	14	0.3	20	0.4
IIId.2	Mixed and unspecified gliomas	225	3.4	227	3.8	179	3.4	185	3.5	162	3.0	174	3.2	169	3.2	171	3.3
IIId.3	Other neuroepithelial tumours	3	0.1	0	–	2	< 0.05	5	0.1	4	0.1	1	< 0.05	6	0.1	17	0.3
IIIe.1	Pituitary adenoma	5	0.1	10	0.1	6	0.1	6	0.1	10	0.2	10	0.2	30	0.5	37	0.6
IIIe.2	Craniopharyngioma	70	1.1	91	1.5	65	1.2	77	1.4	105	1.9	98	1.7	91	1.6	100	1.9
IIIe.3	Pineal parenchymal tumours	25	0.4	31	0.6	22	0.3	17	0.3	16	0.3	23	0.4	24	0.5	22	0.4
IIIe.4	Mixed glial-neuronal tumours	5	0.1	6	0.1	10	0.2	15	0.3	48	0.9	100	1.8	104	1.9	119	2.2
IIIe.5	Meningioma	26	0.4	21	0.3	21	0.3	15	0.3	31	0.5	17	0.3	22	0.4	28	0.5
IIIf	Unspecified tumours	153	2.3	61	1.1	83	1.6	86	1.7	104	1.9	67	1.3	97	1.9	99	1.9
Xa	Germ cell tumours	33	0.5	50	0.8	67	1.2	56	1.1	82	1.5	85	1.5	83	1.5	87	1.6
ICD-O-3 behaviour																	
0, 1	Non-malignant	475	7.3	481	8.2	407	7.5	429	8.3	623	11.5	746	13.6	823	15.5	903	17.2
3	Malignant	1180	18.5	1153	19.8	1078	20.5	1123	21.8	1257	23.4	1163	21.8	1208	23.5	1232	24.1
Total		1655	25.7	1634	28.0	1485	28.1	1552	30.1	1880	34.9	1909	35.4	2031	38.9	2135	41.3

Source: National Registry of Childhood Tumours

The ASR for total intracranial and intraspinal tumours rose from 25.7 per million in 1971–75 to 41.3 per million in 2006–10, an increase of 60% over 35 years. The corresponding increases in age-specific rates (not shown in Table 4) were: age 0, from 20.3 per million in 1971–75 to 42.9 per million in 2006–10, 108%; age 1–4, from 28.5 per million in 1971–75 to 44.5 per million in 2006–10, 56%; age 5–9, from 27.3 per million in 1971–75 to 40.5 per million in 2006–10, 48%; age 10–14, from 22.5 per million in 1971–75 to 38.3 per million in 2006–10, 70%.

**Fig. 1:**
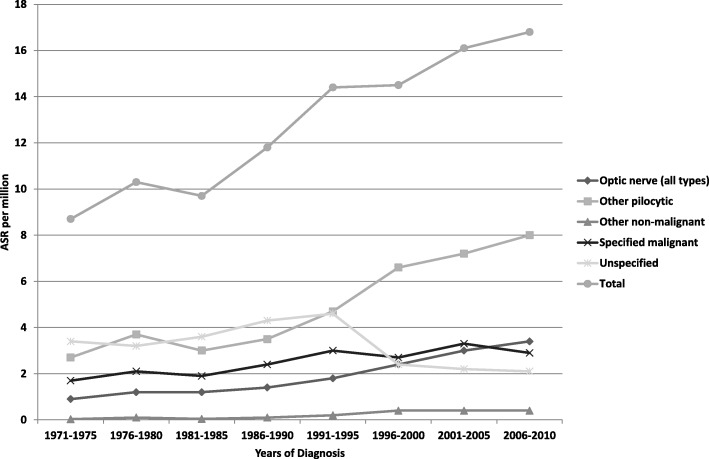
Age-standardised rates of childhood astrocytoma in Great Britain by five-year calendar period of diagnosis, 1971–2010

Incidence patterns over time varied markedly between diagnostic categories. Astrocytoma was the most frequent tumour type throughout, and its ASR rose from 8.7 per million in 1971–75 to 16.8 per million in 2006–10, an increase of 93%. Between the same two periods, the proportional increase was greater for pilocytic astrocytoma (202%, from 2.9 per million to 8.6 per million) than for astrocytomas of other or unspecified subtype (40%, from 5.8 per million to 8.2 per million), and higher for optic nerve astrocytomas (286%, from 0.9 per million to 3.4 per million) than for other sites (72%, from 7.8 per million to 13.4 per million). From 1991 onwards, pilocytic astrocytoma was the most frequent single histological type of tumour (Fig. 1).

Medulloblastoma, mixed and unspecified gliomas, and ependymoma, all had very similar ASRs in 1971–75 and 2006–10, with fluctuating rates in the intervening periods. Among the remaining, rarer categories, some showed little sign of consistent change over time, such as oligodendroglioma and pineal parenchymal tumours. Several others were registered extremely rarely in the earliest years. Neuronal and mixed neuronal-glial tumours accounted for no more than 1% of all tumours before 1991, but their relative frequency rose to 2.6% in 1991–95 and 5.3% in 1996–2010, corresponding to increases in registration rates initially for ganglioglioma and later for DNET and DIA. Some tumours, notably ATRT and pituitary adenoma, were only recorded in substantial numbers in the final decade.

When classified by ICD-O-3 behaviour code, incidence of both non-malignant and malignant tumours increased between 1971 and 75 and 2006–10 (Table 4). The ASR for tumours with non-malignant behaviour code increased by 137%, from 7.3 to 17.2 per million. The corresponding change for malignant tumours was 30%, from 18.5 to 24.1 per million. For all tumours except ICCC-3 IIIb, astrocytoma, the increases in ASR were: non-malignant, 4.4–8.2 per million, 86%, and malignant, 12.7–16.3 per million, 29%.

### Contribution of second primary neoplasms

In 2001–10 there were 44 registrations for CNS tumours as second or later primary neoplasms among children (Table 5). Ependymomas accounted for 2 (5%), astrocytomas for 16 (36%), other gliomas for 5 (11%), embryonal tumours for 9 (20%), meningiomas for 6 (14%), mixed glial-neuronal tumours for 3 (7%), unspecified tumours for 2 (5%) and germ-cell tumours for 1 (2%). There were no choroid plexus tumours, pituitary tumours, craniopharyngiomas or pineal parenchymal tumours. Thirty-four tumours (77%) were in children aged 10–14 years. Second primaries represented 1% of all childhood CNS tumours, but 11% of oligodendrogliomas and meningiomas. The proportion of childhood CNS tumours that were second primaries rose from zero in the first year of life to 0.2% at age 1–4, 0.5% at age 5–9 and 2.5% at age 10–14.

**Table 5:** Intracranial and intraspinal tumours as second or later primary neoplasms among children aged 0–14 years resident in Great Britain, 2001–2010. Numbers of cases by age group, with total numbers of cases as first or only primary neoplasm for comparison

		Second neoplasms by age at diagnosis (years)					First neoplasms
ICCC-3		0	1–4	5–9	10–14	Total	Total
IIIa	Ependymoma & choroid plexus tumours	0	0	0	2	2	402
IIIa.1	Ependymoma	0	0	0	2	2	291
IIIb	Astrocytoma	0	2	1	13	16	1707
IIIc	Embryonal tumours	0	0	2	7	9	717
IIIc.1	Medulloblastoma	0	0	2	0	2	522
IIIc.2	Embryonal CNS tumour NOS	0	0	0	6	6	111
IIIc.4	ATRT	0	0	0	1	1	81
IIId	Other gliomas	0	0	1	4	5	397
IIId.1	Oligodendroglioma	0	0	0	4	4	34
IIId.2	Mixed and unspecified gliomas	0	0	1	0	1	340
IIIe	Other specified tumours	0	1	1	7	9	577
IIIe.4	Mixed glial-neuronal tumours	0	0	1	2	3	223
IIIe.5	Meningioma	0	1	0	5	6	50
IIIf	Unspecified tumours	0	0	1	1	2	196
Xa	Germ cell tumours	0	0	1	0	1	170
Total		0	3	7	34	44	4166

Source: National Registry of Childhood Tumours

## Discussion

The data presented here give up to date estimates of the incidence of intracranial and intraspinal tumours among children in Great Britain. Total annual incidence during 2001–10 was 40.1 per million, equivalent to a risk of 1 in 1678 that a child will be diagnosed with such a tumour before their 15th birthday. About 40% of incidence was accounted for by astrocytomas. The most frequent single histological type of tumour was pilocytic astrocytoma, which accounted for one half of all astrocytomas and 21% of all tumours. If it is assumed that almost all unspecified optic nerve gliomas and astrocytomas in children are actually pilocytic astrocytomas [22, 23], these proportions increase to 65 and 27% respectively. Embryonal tumours, predominantly medulloblastoma, accounted for 17% of all registrations, and ependymomas and other gliomas together for 17%. The remaining 25% consisted of a wide range of other, rarer entities together with a small contribution from tumours of unspecified type.

In addition to providing current estimates of incidence for the most frequent and long-established types of childhood CNS tumour, the data also include reasonable numbers of cases of several tumours that entered ICD-O at the third edition [16]. The most numerous of these is DNET, a low-grade glioneuronal tumour whose incidence increases steadily with age throughout childhood. DNET has similar incidence to ganglioglioma, accounting for about 40% of mixed glial-neuronal tumours and 2% of all CNS tumours.

ATRT is the third most frequent embryonal CNS tumour, after medulloblastoma and embryonal CNS tumour NOS. It predominantly occurs in very early childhood, and in the first year of life its incidence is similar to that of medulloblastoma and embryonal CNS tumour NOS. In the past, most cases would have been diagnosed as medulloblastoma or PNET, which ATRT can resemble morphologically [24]. In a UK series of 42 centrally reviewed cases of embryonal CNS tumour NOS, six (14%) were reclassified as ATRT because of INI1-immunonegativity and presence of rhabdoid cells [25].

Pilomyxoid astrocytoma was formerly regarded as a relatively aggressive variant of pilocytic astrocytoma. In the present data it accounted for 2.2% of all astrocytomas and 4.2% of the total of pilocytic and pilomyxoid astrocytomas. Incidence was highest in the first year of life, when it accounted for 14% of all astrocytomas and 38% of pilocytic and pilomyxoid tumours. These relative frequencies are probably underestimates. In a diagnostic review of all tumours labelled pilocytic or pilomyxoid astrocytoma over a 14-year period at one neurosurgical centre, 9/91 (10%) of all cases and 6/40 (15%) in children aged under 15 had a final diagnosis of pilomyxoid astrocytoma [26].

DIA, sometimes formerly known as desmoplastic infantile ganglioglioma, is also classified as a glioneuronal tumour. As its name suggests, it is overwhelmingly a tumour of very early life. About 5% of all CNS tumours in infants were DIA in the present series. All other tumour types that were new to ICD-O with the third edition [16] or its first revision [19] were extremely rare, with fewer than 2 registrations per year.

The patterns of incidence described here for Britain are broadly similar to those observed in recent national registration data from France [17], the United States [18] and Australia [27] (Table 6). Total age-standardised incidence ranged from 37.5 per million in Australia to 47 per million in the United States. Astrocytoma was everywhere the most common subgroup, accounting for 36–43% of total incidence, and astrocytoma plus other gliomas accounted for 48–54%. Embryonal tumours accounted for 18–20% of total incidence in all four countries.

**Table 6:** Incidence of intracranial and intraspinal tumours among children aged 0–14 years in Great Britain (NRCT), France [17], the United States [18] and Australia [27]. Age-standardised rates (world standard population) per million; rates for United States calculated using case numbers and denominators from Table 8 and Appendix B respectively of reference 18

		Great Britain	France	United States	Australia
ICCC-3		2001–2010	2000–2008	2006–2010	1997–2006
IIIa	Ependymoma & choroid plexus tumours	4.1	3.8	4.3	3.4
IIIa.1	Ependymoma	2.9	2.6	3.0	–
IIIa.2	Choroid plexus tumours	1.2	1.2	1.3	–
IIIb	Astrocytoma	16.4	13.7	17.3	16.2
IIIc	Embryonal tumours	7.1	7.7	8.4	7.2
IIIc.1	Medulloblastoma	5.1	5.4	–	–
IIIc.2	Embryonal CNS tumour NOS	1.1	1.1	–	–
IIIc.4	ATRT	0.9	1.1	–	–
IIId	Other gliomas	3.7	4.9	6.1	4.2
IIId.1	Oligodendroglioma	0.3	1.6	0.5	–
IIIe	Other specified tumours	5.3	6.0	8.2	3.9
IIIe.1	Pituitary adenoma	0.5	0.2	1.7	–
IIIe.2	Craniopharyngioma	1.7	1.8	2.0	–
IIIe.3	Pineal parenchymal tumours	0.5	0.3	0.4	–
IIIe.4	Mixed glial-neuronal tumours	2.1	3.2	3.2	–
IIIe.5	Meningioma	0.4	0.6	0.8	–
IIIf	Unspecified tumours	1.9	0.6	1.7	0.9
Xa	Germ cell tumours	1.5	2.1	1.8	1.8
Total		40.1	38.8	47.0	37.5

**Fig. 2:**
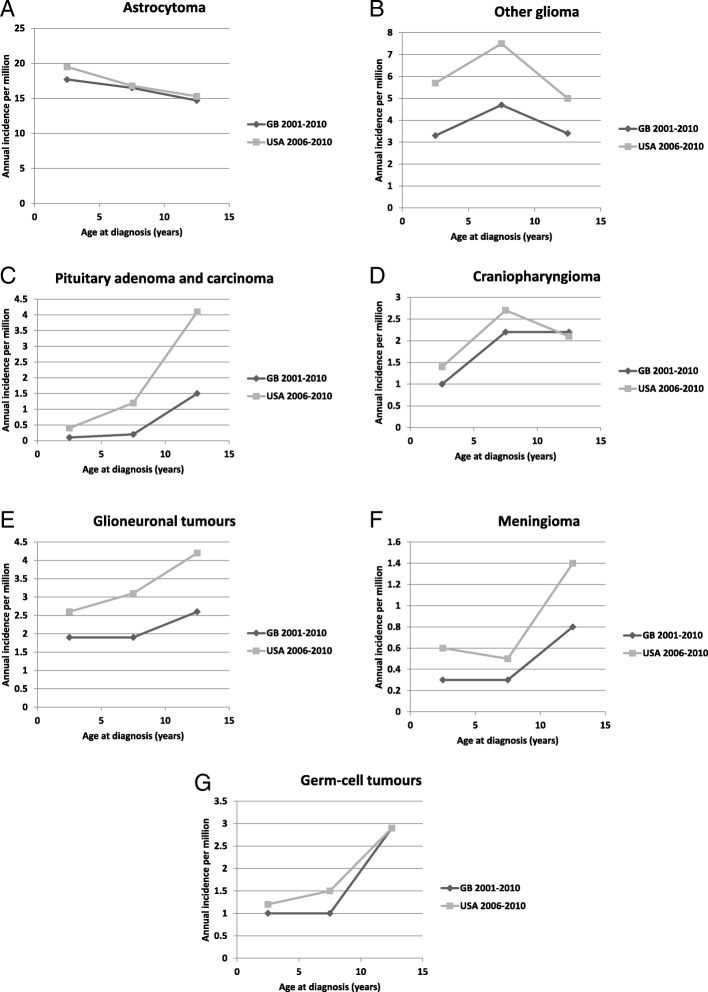
Age-specific rates of selected types of CNS tumours in Great Britain 2001–2010 (present study) and the United States 2006–2010 [18]

The excess in the United States was largely attributable to ICCC-3 subgroups IIId (‘Other gliomas’) and IIIe (‘Other specified tumours’) (Table 6 and Fig. 2). Within the broad category of gliomas, recorded incidence of astrocytoma was 10% higher in the United States at age 0–4 but only 2–4% higher within the 5–14 year age range. Incidence of other gliomas, mainly unspecified, in the United States was about 1.6 times that in Britain; the % MV was not stated [18]. The other categories that had markedly higher rates in the United States were: neuronal/glial tumours, about 1.5 times as frequent as in Britain; meningioma, twice as frequent; and pituitary tumours, about 3.5 times as frequent [18]. It is impossible to know how much of these differences is due to higher levels of ascertainment and how much to earlier diagnosis of predominantly low-grade tumours that can have a protracted natural history. The excess of glio-neuronal tumours was slightly higher at age 5–14 (1.6 times) than at age 0–4 (1.4 times), suggesting that incidence of DNET, in particular, is substantially higher in the United States. The British data excluded second primary tumours, but their inclusion would only have increased the total number of registrations for childhood meningiomas by 12%, a very small part of the difference between the two countries. Proportionally, the largest difference in incidence was for pituitary adenoma, with ASR of 0.5 per million in Britain and 1.7 in the United States. Registration of this tumour was certainly incomplete in Britain in the past. Published case series from two major treatment centres in London contain sufficient information for a case-by-case comparison with registry data. One series [28] included 11 children diagnosed in 1982–96, of whom a maximum of 4 (36%) were registered. The other [29, 30] included 26 children diagnosed in 1983–2008 of whom no more than 4 (15%) were registered. Thus the maximum estimate of the proportion registered from the combined series was only 8/37 (22%). The difference between the two countries in incidence of craniopharyngioma was relatively small but age at diagnosis was younger in the United States (Fig. 2), which may tend to support earlier diagnosis as an explanation for some of the other differences.

In France, incidence of oligodendroglioma was considerably higher than elsewhere and incidence of astrocytoma correspondingly lower, perhaps because French pathologists are more influenced by the St Anne classification in which some diffuse or anaplastic astrocytomas are considered as oligodendrogliomas [31]. Total incidence in Australia was somewhat lower than in Britain, with the deficit accounted for by ICCC-3 subgroups IIIa ependymoma and choroid plexus tumours, IIIe other specified tumours and IIIf unspecified tumours, consistent with under-recording of non-malignant tumours, although it was stated that these were registered in Australia [27].

In the four Nordic countries of mainland Europe during 1985–2006, when incidence of childhood CNS tumours was stable over time, the ASR for ICCC-3 group III (i.e. excluding germ-cell tumours) was 42.0 per million [32], compared with 38.6 per million in Britain during 2001–10. The excess in the Nordic countries was less pronounced at age 10–14 than in the first decade of life, and was concentrated in astrocytoma (ASR 17.9 per million) and unspecified tumours (3.2 per million). In Sweden during 1984–2005, incidence rates were similar to those reported for the combined Nordic countries; optic nerve/chiasm gliomas had an ASR of 3.8 per million and accounted for 17% of astrocytomas, and 67% were diagnosed before age 5 years [33]. In Britain during 2001–10, optic nerve gliomas had an ASR of 3.2 per million and accounted for 18% of astrocytomas; 56% were diagnosed at age under 5 years. The lower ASR and lower proportion diagnosed at young ages indicate that diagnosis of optic nerve gliomas is delayed in Britain compared to Sweden.

The German Childhood Cancer Registry (GCCR) registered 4270 tumours in ICCC-3 Group III and subgroup Xa during 1998–2007 [34]*.* Embryonal tumours accounted for a higher proportion of registrations (21%) than in Britain (17%). Astrocytomas also had a higher relative frequency than in Britain (45% vs 41%), offset by lower relative frequencies of other gliomas (7.5% vs 9.5%) and unspecified tumours (1.4% vs 4.7%). More detailed data on CNS tumours in the GCCR have been published for the periods 1990–9 and 1980–99 [35]. Compared with Britain, Germany had a lower ratio of medulloblastoma to embryonal CNS tumour NOS which may be related to the exceptionally close relationship between the GCCR and national clinical trials. The GCCR also had a lower ratio of pineoblastoma to non-malignant pineal parenchymal tumours, for which it is difficult to suggest a plausible explanation.

Many cancer registries, while generally restricting their coverage to malignant neoplasms, also systematically collect data on non-malignant tumours of certain sites because of their pre-malignant status, or because they are potentially lethal and the distinction from malignant tumours can be difficult and opinion as to malignancy has changed over time. The former category is exemplified by tumours of the bladder and cervix. CNS and other intracranial tumours are the most important example of the latter. Some important childhood tumours of these sites, including craniopharyngioma, have always been classed as non-malignant, and will thus have been consistently excluded from presentations of data that are limited to malignant tumours. Many population-based cancer registries in Europe, and all specialist childhood cancer registries, have traditionally registered all CNS tumours regardless of behaviour. By contrast, most state cancer registries in the United States, notably those affiliated to the Surveillance, Epidemiology and End Results (SEER) Program of the National Cancer Institute, did not systematically record or publish data on non-malignant CNS tumours [18]. However, this gap was filled following the passing of the Benign Brain Tumor Cancer Registries Amendment Act in 2002, with all population-based cancer registries in the United States ascertaining non-malignant CNS tumours diagnosed from 2004 onwards [18]. The Central Brain Tumor Registry of the United States (CBTRUS) has aggregated data from an increasing number of state cancer registries to provide high-quality, population-based statistical data on primary malignant and non-malignant brain and CNS tumours; its report for 2006–10 assembled data from 50 state cancer registries participating in the SEER Program or the National Program of Cancer Registries, representing over 98% of the national population [18]. Many major cancer registries in other world regions that formerly collected only malignant tumours now also register non-malignant CNS tumours, for example the Korea Central Cancer Registry as from 2005 [36].

While non-registration of tumours that have always been regarded as non-malignant leads to underestimates of both incidence and survival for total CNS tumours, the effect is at least reasonably constant over time. Comparability and continuity of data over time are disrupted if the definition of malignancy changes. The data presented here illustrate the severity of the problem when pilocytic astrocytoma, the most frequent of all childhood CNS tumours, undergoes a change of behaviour code. It would not be sufficient to report just the tumours that were coded as malignant in ICD-O-3 because unspecified astrocytoma has always been coded as malignant and it is clear that higher proportions of pilocytic astrocytomas were recorded as unspecified astrocytoma in earlier years. The effect of inclusion/exclusion of pilocytic astrocytoma from survival estimates was illustrated in the EUROCARE-4 study, where 5-year survival for children in Europe with astrocytoma diagnosed in 2000–02 was 63% when tumours specified as pilocytic were excluded and 78% when they were included [37]. In EUROCARE-5, age-standardised 5-year survival for children with malignant astrocytoma defined according to ICD-O-3, so excluding pilocytic astrocytoma, was 64.3% in 1999–2001, 54.5% in 2002–04 and 59.1% in 2005–07 [38]. The decrease in survival after 2002 was probably an artefact of larger numbers of pilocytic astrocytomas being coded as such, and thus excluded, and correspondingly fewer being coded as astrocytoma NOS. Also in EUROCARE-5, 5-year survival for all children with astrocytoma diagnosed in 2000–07 was 80% [39]. For the more numerous specific subtypes, survival ranged from 95 to 100% for pilocytic astrocytoma, optic nerve glioma and SEGA (all WHO Grade I) and 75–85% for fibrillary astrocytoma and pleomorphic xanthoastrocytoma (both Grade II) to 21% for anaplastic astrocytoma (Grade III) and 14% for glioblastoma (Grade IV). For astrocytoma NOS, which accounted for 16% of all astrocytomas and 35% of those with malignant behaviour code, 5-year survival was 74% [39]. This group would have consisted of unknown proportions of diffuse astrocytoma (Grade II) and of tumours of unspecified subtype whose grade was not reported.

The present study concerns childhood tumours but it is equally important that non-malignant tumours diagnosed at older ages are registered. In England during 1995–2003, when pilocytic astrocytoma was still coded as malignant, 17% of CNS tumours diagnosed at age 0–14 were of uncertain, borderline or benign behaviour, compared with 41% at age 15–24 and 42% at age 25–84 [22]. Meningiomas and pituitary tumours, the great majority of which are non-malignant, accounted for 17 and 9% respectively of CNS tumours at age 25–84 during that period [22]. In the United States in 2006–10, non-malignant tumours accounted for about 30% of total age-adjusted CNS tumour incidence in children aged 0–14 years and two thirds of age-adjusted incidence in adults above 20 years of age [18].

In common with many other countries, the British registration data for childhood CNS tumours showed an increase in recorded incidence over time for most of the study period. While there have been major shifts in assignment of specific tumour types to malignant or non-malignant categories over the years, no entities have switched from neoplastic to non-neoplastic or vice versa in the ICD. Therefore, changes in recorded incidence of CNS tumours overall or of any subtype must be due to changes in underlying risk, changes in the proportion actually diagnosed before the 15th birthday, or changes in completeness of ascertainment of diagnosed tumours by the registration system. A study of time trends in registration rates for all types of childhood cancer in Britain between 1966 and 2005 found that changes in incidence of CNS tumours considered as a single group correlated well with contemporaneous changes in diagnostic and registration practice [40]. It did not, however, seek to explain changes over time in rates for specific tumour types or in the ratio of rates for one type to another. Some tumour types were registered hardly ever, if at all, in the earliest years, and only in appreciable numbers later in the study period. This most obviously applies to embryonal CNS tumour NOS (from the 1980s onwards) and ATRT (from the mid-1990s). Registrations for mixed glial-neuronal tumours were also extremely rare in the 1970s but the numbers increased steadily through the following decades as ganglioglioma was more frequently registered in the 1980s, followed by DIA and DNET from the mid-1990s, the latter probably resulting from increasing surgery for epilepsy. The increase in germ-cell tumours from the 1980s was partly offset by a decline in pineal parenchymal tumours, perhaps because some cases of germinoma would have been registered as pinealoma in the early years. Pituitary adenoma is known to have been under-registered, as discussed above. The relative contribution of earlier diagnosis and improved ascertainment to the steep increase in recorded incidence is unknown. Even in the most recent years, incidence was still markedly lower than in the United States. The increase in choroid plexus tumours is perhaps hardest to account for. Choroid plexus papilloma and carcinoma were both well-defined and described before the 1970s, and there was no clear trend over time in the ratio of papilloma to carcinoma.

Within the broad group of astrocytomas, optic nerve tumours increased in incidence more steeply than those of other sites (Table 4). Moreover, the proportions of optic nerve gliomas that were diagnosed before the age of 5 years were 40% in 1971–80, 62% in 1981–90, 53% in 1991–2000 and 56% in 2001–10, consistent with a tendency to earlier diagnosis since about 1980. Nevertheless, as discussed above, the data presented here are compatible with diagnosis of lower grade CNS tumours occurring later in Britain than in some other countries. Earlier diagnosis is important because, even if overall survival is unaffected, disease progression is likely to be less severe [41]. The ‘HeadSmart: Be Brain Tumour Aware’ campaign improved awareness of childhood brain tumours in the UK and was associated with a 7-week reduction in median total diagnostic interval [42]. Other factors that could adversely affect time to diagnosis include availability of magnetic resonance imaging scanners, for which UK provision per million population has been lower than in many other OECD countries [43].

This study has several considerable strengths. The results are derived from a very large population-based data set. Non-malignant tumours were eligible and routinely included throughout the study period. Access to diagnostic reports for the great majority of cases allowed a high degree of specificity in coding, including the possibility of manual recoding of tumours when updating to successive revisions of the coding system. Even so, in some cases it was impossible to obtain evidence regarding whether a tumour was microscopically verified, so the MV percentages in Table 3 should be regarded as minimum estimates.

The most important limitation is that central diagnostic review is impracticable for such a large national series, hence accuracy of diagnosis is an obvious concern. During 2001–10, childhood brain tumours were diagnosed pathologically in 21 hospitals throughout Britain, and the number in earlier years was much greater (data not shown). The potential effects are illustrated by the results of pathology review in a Children’s Cancer Group study of high-grade gliomas in North America, in which only 69% of tumours with an institutional diagnosis of high-grade glioma were confirmed as high-grade glioma by the review panel, and levels of agreement for specific tumour types such as anaplastic astrocytoma and glioblastoma multiforme were even lower [44]. In a UK study of low-grade glioma, however, only 33/713 (5%) of those with central histology review were excluded because of ineligible or inconclusive histology or no evidence of tumour [45]. In international trials where UK centres contributed the majority of patients, 12% of tumours with a local diagnosis of supratentorial embryonal CNS tumour NOS were found not to be embryonal CNS tumour NOS on review [46] and 13% of children under 3 years of age with a tumour other than ependymoma had their diagnosis changed on review [47]. The proportions of tumours such as craniopharyngioma for which histological subtype was recorded was disappointingly low. In some cases this was because a copy of the original pathology report was not obtained, but there were certainly some cases for which the report did not specify a subtype (data not shown).

While this study documents the evolving patterns of recorded incidence of childhood CNS tumours in successive quinquennia over a 40-year period, it is not a formal analysis of time trends and confidence intervals for rates are not provided.

In the absence of central review, the data presented here give the most accurate indication of childhood CNS tumour incidence based on histological criteria. Many tumours, however, would likely be reclassified on review using the genotypic criteria of WHO 2016, e.g. some cases of brainstem glioma or diffuse intrinsic pontine glioma could be redefined as diffuse midline glioma H3 K27 M-mutant [13]. Universal application of WHO 2016 criteria would inevitably modify the demographics of incidence by tumour type, and the use of these less subjective molecular criteria may also reduce variation in tumour diagnoses between institutions and geographic areas. However, definitive population-level description of childhood CNS tumour incidence according to WHO 2016 will only be possible when several years’ registration data according to the new criteria become available.

## Conclusions

Total incidence of childhood CNS tumours in Britain in the first decade of the new millennium was fairly similar to that in other large western countries. Deficits of some, predominantly low-grade, tumours or differences in their age distribution compared with the United States and the Nordic countries are compatible with delayed diagnosis in Britain. Complete registration of all types of CNS tumour, regardless of behaviour, is essential for assessing burden of disease and changes over time in incidence and survival. This is particularly important for pilocytic astrocytoma, the most common of all childhood CNS tumours, because of the recent change in its behaviour code from malignant to non-malignant and changes over time in the proportion of astrocytomas for which subtype has been specified.

## Abbreviations

ASR: Age-standardised rate
ATRT: Atypical teratoid/rhabdoid tumour
CBTRUS: Central Brain Tumor Registry of the United States
DIA: Desmoplastic infantile astrocytoma
DNET: Dysembryoplastic neuroepithelial tumour
GCCR: German Childhood Cancer Registry
ICCC-3: International Classification of Childhood Cancer, Third Edition
ICD-O-3: International Classification of Diseases for Oncology, Third Revision
MV: Microscopically verified
NRCT: National Registry of Childhood Tumours
PNET: Primitive Neuroectodermal Tumour
SEER: Surveillance, Epidemiology and End Results
SEGA: Subependymal giant cell astrocytoma
